# Interaction of childhood abuse and depressive symptoms on cortical thickness: a general population study

**DOI:** 10.1007/s00406-022-01387-8

**Published:** 2022-02-25

**Authors:** Sara Voss, Stefan Frenzel, Johanna Klinger-König, Deborah Janowitz, Katharina Wittfeld, Robin Bülow, Henry Völzke, Hans J. Grabe

**Affiliations:** 1 Department of Psychiatry and Psychotherapy, University Medicine Greifswald, Ellernholzstraße 1-2, 17475 Greifswald, Germany; 2 Clinical Centre for Psychiatry und Psychotherapy, Site West, Stralsund, Germany; 3 German Centre for Neurodegenerative Diseases (DZNE), Site Rostock/Greifswald, Greifswald, Germany; 4 Institute for Diagnostic Radiology und Neuroradiology, University Medicine Greifswald, Greifswald, Germany; 5 Institute for Community Medicine, SHIP/Clinical-Epidemiological Research, University Medicine Greifswald, Greifswald, Germany

**Keywords:** Cortical thickness, Childhood abuse, Depressive symptoms, Resilience

## Abstract

Childhood abuse was inconsistently related to whole-brain cortical thickness in former studies. However, both childhood abuse and cortical thickness have been associated with depressive symptoms. We hypothesised that childhood abuse moderates the association between depressive symptoms and cortical thickness. In 1551 individuals of the general population, associations between whole-brain cortical thickness and the interaction of childhood abuse (emotional, physical, and sexual) and depressive symptoms were analysed using an ANCOVA. Linear regression analyses were used to estimate the same effect on the cortical thickness of 34 separate regions (Desikan-Killiany-atlas). A significant interaction effect of childhood abuse and depressive symptoms was observed for whole-brain cortical thickness (*F*(2, 1534) = 5.28, *p* = 0.007). A thinner cortex was associated with depressive symptoms in abused (*t* value = 2.78, *p* = 0.025) but not in non-abused participants (*t* value = − 1.50, *p* = 0.224). Focussing on non-depressed participants, a thicker whole-brain cortex was found in abused compared to non-abused participants (*t* value = − 2.79, *p* = 0.025). Similar interaction effects were observed in 12 out of 34 cortical regions. Our results suggest that childhood abuse is associated with reduced cortical thickness in subjects with depressive symptoms. In abused subjects without depressive symptoms, larger cortical thickness might act compensatory and thus reflect resilience against depressive symptoms.

**Supplementary Information:**

The online version contains supplementary material available at 10.1007/s00406-022-01387-8.

## Introduction

The *American Professional Society on the Abuse of Children* (APSAC) defines childhood abuse as “words or overt actions that cause harm, potential harm, or threat of harm to a child” [1] and differentiates between emotional, physical, and sexual abuse. In Germany, the prevalence of emotional abuse was estimated at 6.5%, physical abuse at 6.7%, and 7.6% at sexual abuse [2].

Childhood abuse initiates physiological and psychological stress-response systems [3], contributing to long-term changes in epigenetic processes, gene regulation, and brain development [4, 5]. Thus, childhood abuse is associated with higher lifetime risks of somatic illnesses such as metabolic syndrome, cardiovascular and respiratory disorders [6]. Additionally, childhood abuse is associated with the development of mental diseases such as major depressive disorder (MDD) [7, 8] and posttraumatic stress disorder [8]. Therefore, the genesis of mental diseases is, besides genetic factors, influenced by environmental aspects such as childhood abuse, probably through epigenetic mechanisms [9]. Hence, structural alterations may precede the onset of the mental disease [9]. In contrast, Teicher et al. [5] stated in their review in *Nature Reviews Neuroscience* that certain alterations in maltreated subjects are *not* linked to psychopathology and that maltreated subjects could also differ in brain changes which help them compensate [5]. Thus, understanding how childhood abuse increases the risk of physical and mental diseases and, thus, enabling the implementation of focused prevention programs to reduce pathological consequences is of high demand [5]. The exact mechanisms behind these associations are still elusive.

Several studies suggest that different patterns of brain alterations are associated with different types of maltreatment. Thus, parental verbal abuse was linked to a reduction in the auditory cortex [10] and sexual abuse was associated with a reduced extended genital representation in the sensomotoric cortex [11]. On the other hand, social deprivation was associated with reductions in regions of the association cortex processing social stimuli [12]. To address these findings, we focussed only on the consequences of childhood abuse on the cortex.

More recently, Tozzi et al. [13] analysed interaction effects of severity and type of childhood maltreatment (abuse or neglect) and MDD on cortical thickness in 3,872 participants within the ENIGMA consortium [13]. They found associations of childhood maltreatment severity with reductions of thinner cortices in the banks of the superior temporal sulcus and supramarginal gyrus [13]. However, they did neither find an interaction effect between childhood maltreatment and MDD, nor a main effect of MDD on cortical thickness. Within the ENIGMA consortium, Schmaal et al. [14] analysed the cortical thickness in 1,902 MDD patients and 7658 controls. They found thinner cortices in MDD patients in the temporal and frontal lobes, including the orbitofrontal cortex [14]. Further, Lim et al. [15] analysed a youth sample (excluding sexual abuse) and found lower cortical thickness in abused participants with psychiatric comorbidities in comparison to healthy non-abused participants. Given that childhood abuse is a crucial risk factor for the development of MDD, it is challenging to disentangle the contribution of both conditions to the cortical thickness [15]. Former findings show divergent results on how depression and childhood abuse affect the cortex. Investigating if and how the interaction of depression and childhood abuse might explain some of these differential outcomes, is a relevant concern.

Although the ENIGMA studies investigating the childhood-maltreatment-MDD-interaction on cortical regions analysed large sample sizes [13], there are limitations essential to consider: covariates such as level of education, alcohol consumption, smoking behaviour, and obesity were missing. However, as these parameters are frequently associated with childhood abuse, MDD, and cortical thickness, they could act as confounders [16, 17]. Further, the inclusion of many different samples increased the heterogeneity of the overall sample which might have lowered the effect sizes [18].

To address these limitations, we used a sample of 1,551 participants from the population-based Study of Health in Pomerania (SHIP) to analyse the interaction of childhood abuse and depressive symptoms on cortical thickness. We focused on current depressive symptoms, which were associated with childhood maltreatment in former studies [19, 20]. More precisely, this study regressed whole-brain cortical thickness in addition to the cortical thickness of 34 single cortical regions on the interaction effect between childhood abuse (yes/no) and depressive symptoms (none/mild/moderate to severe). Previous research revealed differential results on how depression and childhood abuse affect the cortex. We hypothesise that the interaction of childhood abuse and depressive symptoms is associated with the cortical thickness and might explain some of the diverse results.

## Methods

### Sample

SHIP is a population-based cohort study [21]. Local registries randomly drew the study sample of West Pomerania between 2008 and 2011 (SHIP-Trend-0: *N* = 4420). For more details see Völzke et al. [21]. SHIP-Trend-0 participants free of exclusion criteria (e.g. cardiac pacemakers, pregnancy) were invited for a whole-body magnetic resonance imaging (MRI) [22]. MRI of the head was available for 2,154 participants of SHIP-Trend-0 [23]. MRI scans of sufficient quality were available for 1,986 participants (see subdivision *Magnetic Resonance Imaging* for the detailed selection process). This study design excluded participants from the following analyses if the information on depressive symptoms (*n* = 60) or childhood abuse (*n* = 138) was missing or due to incomplete covariate data (*n* = 22; see subdivision *Statistical Analyses*). Finally, as we aimed to compare participants without any history of abuse or neglect with abused participants, we excluded participants if they reported an experience of neglect, but not abuse (*n* = 215). Thus, our final sample comprised 1,551 participants.

The institutional review board of the University Greifswald approved SHIP. Examinations and assessments have been conducted by the declaration of Helsinki, including written informed consent.

### Measures

#### Interview

Sociodemographic data including age, sex, education level, smoking behaviour, intake of antidepressants (yes/no) and alcohol consumption was collected via a computer-assisted face-to-face interview. According to the German school system, educational level was divided into < 10 years (low), = 10 years (medium), and > 10 years of school education (high). SHIP-Trend-0 categorised smoking behaviour into never, ex-, and current smokers. Alcohol consumption was defined as the mean intake of gram ethanol per day over the past 30 days according to Baumeister et al. [24]. Afterwards, participants underwent a physical examination including the measurement of the waist circumference, weight, and height. The examiner measured the waist circumference with the participant standing on both feet to the nearest 0.1 cm. Weight was measured in light clothes to the nearest 0.1 kg. Height was measured to the nearest 1 cm. Participants were asked to report the medication used during the past 7 days and to bring their package containers. Medication was categorised according to the ATC-Index [25]. Antidepressants were defined as ATC codes N06A.

#### Childhood abuse

To enquire about the exposure to childhood abuse, this study used the German version of the Childhood-Trauma-Questionnaire (CTQ) [26, 27]. The CTQ is a 28-item questionnaire assessing maltreatment in childhood and youth [27, 28]. The CTQ measures different exposures of maltreatment in five subscales: emotional, sexual, and physical abuse, emotional and physical neglect. Participants rated each item on a 5-point Likert scale, from “not experienced” to “very often experienced”. The CTQ is an established instrument with decent reported validity (depression: *r* = 0.36, anxiety: *r* = 0.40, health: *r* = − 0.23) and reliability (emotional abuse: Cronbach’s *α* = 0.87, physical abuse: *α* = 0.80, sexual abuse: *α* = 0.89) with a Cronbach’s *α* of 0.94 for the whole questionnaire. For an overview of the German version see Klinitzke et al. [29]. Note again that, as we aimed to compare participants free of moderate to severe childhood maltreatment with abused participants, we excluded participants who exclusively reported emotional and/or physical neglect (emotional neglect ≥ 15; physical neglect ≥ 10) from the study (see section *Sample*). This exclusion criterion, however, did not take effect if participants reported co-experience of abuse and neglect. A dichotomized variable of childhood abuse was generated including sexual, physical, and emotional abuse. This study categorised participants to the abused group if they reached the defined cut-off score for moderate abuse in at least one of the three subscales (sexual abuse ≥ 8; emotional abuse ≥ 13; physical abuse ≥ 10) [27]. Participants were categorised to the non-abused group when they did neither score on the abuse nor the neglect subscales.

#### Depressive symptoms

To enquire depressive symptoms, this study used the Patient-Health-Questionnaire-9 (PHQ-9) [30]. The PHQ-9 assesses the existence of depressive symptoms with nine items, based on the nine criteria for MDD of the Diagnostic and Statistical Manual of Mental Disorders 4th Revision (DSM-IV). A 4-point Likert scale assesses the symptoms from having “no symptoms” to “symptoms every day”. The PHQ-9 has a suitable internal consistency with a Cronbach’s α of 0.88 [31]. Based on the summary score of the PHQ-9, this study design assorted participants into three groups according to the criteria of Kroenke et al. (2002): no depressive (≤ 4), mild depressive (5–9), and moderate to severe depressive symptoms (≥ 10).

### Magnetic resonance imaging (MRI)

MRI of the head was available for 2154 participants of SHIP-Trend-0 [23]. A Siemens Magnetom Avanto scanner acquired T1-weighted images with following parameters: field strength = 1.5 T, orientation = axial plane, repetition time = 1900 ms, echo time = 3.37 ms, flip angle = 15°, slice thickness = 1 mm, and resolution 1 mm × 1 mm. Three scans were eliminated due to poor quality (strong frontal darkening). In addition, 100 scans were excluded due to structural abnormalities (e.g., tumours, cysts) and cerebral stroke. The image segmentation pipeline failed to process four scans. We excluded further 61 participants which were categorised as outliers in various cortical thickness regions (see below), resulting in an imaging sample of 1986 participants.

### Parcellation of the cerebral cortex

The FreeSurfer image analysis suite (version 5.3) performed the cortical reconstruction and volumetric segmentation. This application suite is documented and freely available for download online (http://surfer.nmr.mgh.harvard.edu).

Briefly, this process included the removal of non-brain tissue using a hybrid watershed/surface deformation procedure [32], automated Talairach transformation, intensity normalization [33], tessellation of the grey/white matter boundary, and automated topology correction [34, 35]. Further, surface deformation follows intensity gradients to optimally place the grey/white and grey/cerebrospinal fluid borders at the location where the greatest shift in intensity defines the transition to the other tissue class [36–38].

Individual cortical models were registered to a spherical atlas [39] and parcelled into 68 units with respect to gyral and sulcal structure [40, 41]. Average thickness of each region was computed. Statistical quality control was performed by excluding cases with thickness values higher/lower than four standard deviations from the whole sample mean after adjustment for age, sex, body height, and the interaction of age with sex. 61 participants were excluded based on this criterion.

Cortical thicknesses were averaged across both hemispheres resulting in 34 brain region parameters. Besides, whole-brain cortical thickness was defined as the average of the thicknesses of the left and right hemisphere. The estimated intracranial volume (ICV) was generated by FreeSurfer.

### Statistical analysis

All analyses were adjusted for sex, age, age^2^, sex-age-interaction, estimated intracranial volume (ICV), educational level, alcohol consumption, smoking behaviour, waist-to-height ratio, and body mass index (BMI).

To analyse the main and interaction effects of childhood abuse (yes/no) and depressive symptoms (none/mild/moderate to severe) on whole-brain cortical thickness, we implemented three models for an analysis of variance with covariates (ANCOVA) with whole-brain cortical thickness as the dependent variable. Two models were implemented with the confounding variables and either childhood abuse or depressive symptoms as independent variable. The third model included the interaction term childhood abuse*depression. The same analysis model was conducted with depressive symptoms as a continuous variable in a linear regression by using the sum score of the PHQ-9 (see SI). In an additional model, we included the intake of antidepressants (yes/no) as a covariate in addition to the other covariates mentioned above (see SI). Levene’s Test of Equality estimated the variance homogeneity. The overall test on whole-brain cortical thickness tested with a significance level of *p* < 0.05. Afterwards, 9 post-hoc tests were calculated for the interaction groups using the estimated marginal means. Therefore, we computed pairwise comparisons between the abuse groups at each level of depression and vice versa. These tests were adjusted by sex, age, age^2^, sex-age-interaction (sex × age), BMI, waist-to-height ratio, alcohol consumption, educational level, smoking, and ICV. Educational level and smoking were dummy coded and, thus, represented by two dummy variables, respectively. We used the false discovery rate (FDR) method of Benjamini and Hochberg [42] to adjust the *p* values for multiple testing for the 9 post-hoc pairwise comparisons.

To investigate which brain regions are affected by the interaction of childhood abuse and depressive symptoms, the cortical thickness of 34 segmented cortical regions was examined in separate linear regression analyses. We implemented the childhood abuse*depression interaction term as the independent variable and each cortical region separately as the dependent variable. These tests were adjusted by sex, age, age^2^, sex-age-interaction, BMI, waist-to-height ratio, alcohol consumption, educational level (dummy coded), smoking (dummy coded), and ICV. To correct for multiple testing, FDR correction was used for 34 linear regression analyses.

Descriptive statistics revealed differences in sex proportions and depression level (Table 1) between the abuse and non-abuse group. Thus, abused participants (*n* = 120) were matched 1:1 with non-abused participants (*n* = 120) by sex (male, female), depressive symptoms (using the categories: non, mild, or moderate to severe), and age. We matched for age to adjust for possible age-education-interactions. Nearest neighbour matching was accomplished with the package *MatchIt*. Descriptive statistics of the matched sample are presented in Table SI2. We recalculated all analyses with the matched sample as sensitivity analyses.

**Table 1:** Characteristics of the study sample

	All subjects (*N* = 1551)	Abuse (*n* = 122)	No abuse (*n* = 1429)	*p* value^a^
Sex (female), *n* (%)	834 (54%)	89 (73%)	745 (52%)	< 0.001
Age (years), M ± SD (age range: 21–82)	50 ± 14	50 ± 13	50 ± 14	0.908
BMI, M ± SD	27 ± 4.4	28 ± 4.9	27 ± 4.4	0.591
Educational level				0.294
< 10 years, *n* (%)	167 (11%)	17 (14%)	150 (10%)	
= 10 years, *n* (%)	870 (56%)	71 (58%)	799 (56%)	
> 10 years, *n* (%)	514 (33%)	34 (28%)	480 (34%)	
Alcohol (g/day), M ± SD	8 ± 11	6 ± 8.7	8 ± 11	0.036
Smoking				0.264
Never smoker, *n* (%)	620 (40%)	41 (34%)	579 (41%)	
Ex-smoker, *n* (%)	559 (36%)	46 (38%)	513 (36%)	
Current smoker, *N* (%)	372 (24%)	35 (29%)	337 (24%)	
ICV (dm^3^), *M* ± *SD*	1.587 ± 0.16	1.555 ± 0.17	1.590 ± 0.16	0.019
Depressive symptoms				< 0.001
No, *n* (%)	1064 (69%)	55 (45%)	1009 (71%)	
Mild, *n* (%)	397 (26%)	44 (36%)	353 (25%)	
Moderate to severe, *n* (%)	90 (6%)	23 (19%)	67 (5%)	
PHQ-9 (summary), M ± SD	3.7 ± 3.5	6.6 ± 5.4	3.5 ± 3.1	< 0.001
Neglect (yes), *n* (%)	72 (5%)	72 (59%)	0 (0%)	< 0.001
CTQ (summary), M ± SD	32 ± 9.1	54 ± 17	30 ± 4.1	< 0.001
*Abuse severity (summary) M ± SD*	17 ± 4.8	30 ± 9.4	16 ± 1.6	< 0.001
Abuse Categories				
Emotional Abuse (yes), *n* (%)	61 (4%)	61 (50%)	–	–
Physical Abuse (yes), n (%)	62 (4%)	62 (52%)	–	–
Sexual Abuse (yes), *n* (%)	52 (3%)	52 (43%)	–	–

*PHQ-9* Patient-Health-Questionnaire-9; *CTQ* Childhood-Traumatisation-Questionnaire; *BMI* Body Mass Index; *ICV* Intracranial volume

^a^According to one-way ANOVA for continuous or *χ*^2^ tests for categorical variables to check for possible group differences

A *p* value of < 0.05 was considered statistically significant. All statistical analysis were performed using R version 3.6.2 [43].

## Results

### Demographics

Descriptive statistics are summarised in Table 1. Overall, 1,429 participants reported no exposure to any childhood maltreatment, 122 (8.5%) experienced at least one type of childhood abuse. In the whole sample, 11.0% of the female population reported childhood abuse, thus women were significantly more often affected by childhood abuse than men (4.6%; *χ*^2^ = 18.77, *p* < 0.001). Further, 1,064 participants (68.6%) reported no current depressive symptoms, whereas 397 participants (25.6%) reported mild, and 90 participants (5.8%) reported moderate to severe symptom severity. The participants of the group allocation (abuse: yes/no) did not vary regarding age, waist circumference, smoking, BMI, and educational level. However, there were substantial variations across the two groups in the distribution of sexes (*χ*^2^ = 18.77, *p* < 0.001), ICV (*F*(2, 1548) = 5.47, *p* = 0.019), alcohol consumption (*F*(2, 1548) = 4.41, *p* = 0.036), and in the frequency of the depressive categories (*χ*^2^ = 55.21, *p* < 0.001). Descriptive characteristics of the study sample ordered by the severity of depressive symptoms are displayed in Table SI1.

Thus, to consider these differences in the analyses more clearly, a subsample matched for sex, depression level, and age was extracted. Table SI2 summarizes the descriptive statistics for the matched sample in the supplementary information. In the matched sample, the two abuse-groups did no longer vary in the distribution of sexes, ICV, and the depression level (non, mild, moderate to severe) validating the matching process.

### Effects of childhood abuse and depressive symptoms on whole-brain cortical thickness

The Levene’s-Test was non-significant for the ANCOVA analysis indicating no violation of variance homogeneity. Analysing the influence of childhood abuse on whole-brain cortical thickness, we found no significant effects (*F*(1, 1534) = 1.64; *p* = 0.201). Further, no significant effects were observed for depressive symptoms (*F*(1, 1534) = 0.08; *p* = 0.925). However, there was a statistically significant two-way interaction between childhood abuse and current depressive symptoms on whole-brain cortical thickness (*F*(2, 1534) = 5.28, *p* = 0.007). In addition, there was also a statistically significant two-way interaction between childhood abuse and the continuous variable for depressive symptoms on whole-brain cortical thickness (*F*(2, 1534) = − 2.97, *p* = 0.003).

**Fig. 1:**
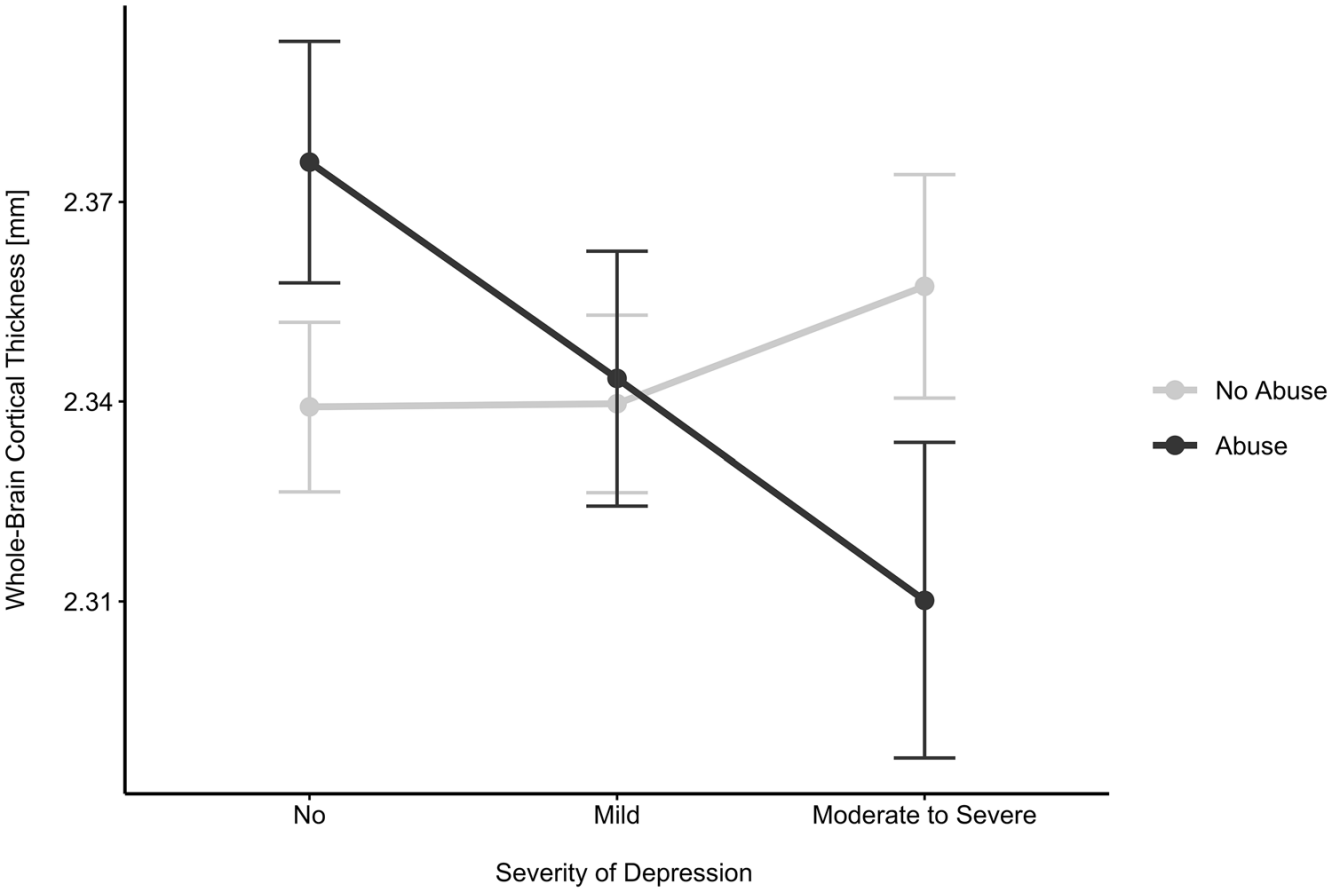
Line plot showing pairwise comparisons between groups using the estimated marginal means and standard errors of whole-brain cortical thickness across all levels of childhood abuse and depression. In the abused-group (black line), participants with moderate to severe depressive symptoms had significantly thinner whole-brain cortices than non-depressed subjects (*p* = 0.025). In the non-depressed (left), whole-brain cortical thickness was larger in abused compared to non-abused participants (*p* = 0.025). See Table SI3 for further information

To investigate this interaction effect in more detail, post-hoc pairwise comparisons were calculated using the estimated marginal means (see Fig. 1 and Table SI3). We adjusted all nine post-hoc comparisons with the false discovery rate method of Benjamini and Hochberg.

Pairwise comparisons were computed between the abuse groups (yes/no) at each level of depression (non, mild, and moderate to severe) and vice versa. In abused participants, we found a statistically significant difference between non-depressed participants and participants with moderate to severe depressive symptoms (*t* value = 2.78, *p*_FDR_ = 0.025). However, there was no significant difference between non-depressed and mildly depressed participants (*t* value = 1.68, *p*_FDR_ = 0.209), nor between mildly depressed and moderately to severe depressed participants (*t* value = 1.36, *p*_FDR_ = 0.224). In contrast, in non-abused participants, no significant differences reached the level of significance across the factor of depression.

In non-depressed participants, we found significant differences between abused and non-abused participants (*t*-value = − 2.79, *p*_*FDR*_ = 0.025). In contrast, childhood abuse had no significant effect in mildly depressed (*t* value = − 0.25, *p*_FDR_ = 0.904), nor moderate to severely depressed participants (*t* value = 2.052, *p*_FDR_ = 0.121).

Using the matched sample (*N* = 240), we recalculated all results. The two-way interaction between childhood abuse and depressive symptoms remained significant (*F*(2, 223) = 4.40, *p*_FDR_ = 0.013). Post-hoc pairwise comparisons revealed statistically significant differences in the abuse group between non-depressed participants and participants with moderate to severe depressive symptoms (*t* value = 3.34; *p*_FDR_ = 0.009) (Fig. SI1).

### Effects of childhood abuse and depressive symptoms on regional cortical thickness

**Fig. 2:**
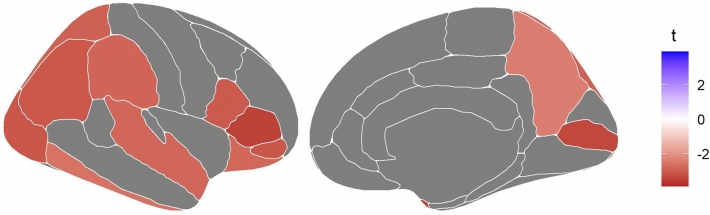
Associations between cortical thickness in 34 cortical regions and the interaction of depressive symptoms and childhood abuse. Colours represent exclusively significant t-values from linear regressions with the brain regions as the dependent variable and adjusted for sex, age, age-sex-interaction, age^2^, ICV, education, alcohol consumption, smoking, BMI, and waist-to-height ratio

To investigate region-specific effects, we tested the interaction of childhood abuse and depressive symptoms on the cortical thicknesses of 34 cortical regions, separately. Table 2 and Fig. 2 present the results**.**

**Table 2:** Associations of the interaction of childhood abuse (CA) and depressive symptoms (categorial variable) (Depr) with regional cortical thickness

Cortical Structure	Interaction Depr X CA	FDR *p* value
Banks of the superior temporal sulcus	− 2.12	0.064
Caudal anterior cingulate	− 0.25	0.818
Caudal middle frontal	− 1.26	0.261
Cuneus	− 1.66	0.137
Entorhinal	0.23	0.818
Frontal pole	− 1.33	0.242
Fusiform	− 1.75	0.118
Inferior parietal	− 3.07	0.010*
Inferior temporal	− 2.60	0.028*
Insula	− 1.92	0.089
Isthmus cingulate	− 1.21	0.274
Lateral occipital	− 3.11	0.010*
Lateral orbitofrontal	− 2.80	0.016*
Lingual	− 2.22	0.062
Medial orbitofrontal	− 2.17	0.062
Middle temporal	− 2.19	0.062
Paracentral	− 1.04	0.352
Parahippocampal	− 2.08	0.068
Pars opercularis	− 3.04	0.010*
Pars orbitalis	− 3.10	0.010*
Pars triangularis	− 3.46	0.010*
Pericalcarine	− 3.37	0.010*
Postcentral	− 2.15	0.062
Posterior cingulate	− 1.01	0.355
Precentral	− 1.35	0.242
Precuneus	− 2.39	0.045*
Rostral anterior cingulate	− 0.76	0.485
Rostral middle frontal	− 2.05	0.070
Superior frontal	− 0.75	0.485
Superior parietal	− 2.90	0.015*
Superior temporal	− 2.81	0.016*
Supramarginal	− 2.85	0.015*
Temporal pole	− 3.46	0.010*
Transverse temporal	− 1.90	0.090

Linear regression analyses were adjusted for sex, age, age-sex-interaction, age^2^, estimated intracranial volume, education, alcohol consumption, smoking, BMI, and waist-to-height ratio. FDR-*p*-values are displayed. Significant FDR-*p*-values are highlighted

After the FDR-correction for 34 cortical regions, the interaction effect was significantly associated with cortical thickness in 12 cortical regions within the inferior frontalis gyrus (pars opercularis, pars orbitalis, pars triangularis), supramarginal gyrus, inferior temporal gyrus, lateral occipital gyrus, lateral orbitofrontal cortex, pericalcarine cortex, precuneus, superior parietal lobule, superior temporal gyrus, and temporal pole (*p* < 0.05 after FDR correction). These cortical regions replicated the interaction effect as seen in the analysis of the whole-brain thickness; cortical thickness is most reduced in the depressed participants with a history of abuse.

Using the matched sample, significant interaction effects were observed in the inferior frontalis gyrus (including the pars opercularis, and the pars triangularis), the inferior parietal gyrus, supramarginal gyrus, and temporal pole, highlighting these regions despite the smaller sample size (see Table SI4).

## Discussion

To deepen the understanding of the interplay between childhood abuse and depressive symptoms, this study investigated the interaction of both risk factors on cortical thickness in a large population-based sample. This study adds essential results to the research domain examining the effects of childhood abuse on mental health by focusing exclusively on childhood abuse interacting with depressive symptoms, extending the commonly used covariate list, and analysing both healthy and depressed, in addition to abused and non-abused participants.

### The interaction of childhood abuse and depressive symptoms on whole-brain cortical thickness

In the present study, only in abused participants, lower whole-brain cortical thickness was associated with more severe depressive symptoms.

Based on our results, studies analysing the effects of childhood abuse on cortical thickness should not neglect the impact of depressive symptoms. Against our assumption, abused participants without depressive symptoms showed an increased whole-brain cortical thickness in contrast to non-depressed and non-abused participants in our sample. This finding is in congruence with a recent study reporting increased cortical thickness in non-depressed (compared to depressed), abused participants in the orbitofrontal cortex [44]. Here, we found a significant interaction in the lateral orbitofrontal gyri. Interestingly, Habets et al. [45] found a similar effect in patients with schizophrenia and their healthy siblings, whereas both experienced childhood maltreatment. They found a reduction in cortical thickness in the patient group and an increase of cortical thickness in the healthy sibling group. These studies show, consistent to our data, that comparing healthy participants without a history of childhood abuse with both abused and depressed participants does display only a small reflection of the whole interaction context.

On the other hand, the variable of childhood abuse appears to be a relevant moderator when analysing the effects of depressive symptoms on cortical thickness. Thus, studies analysing the association between depression and cortical thickness might be more likely to find significant results if they do not control for childhood abuse or have a high proportion of abused participants, which might be especially true for patient samples [5]. Our results suggest that depressive symptoms alone, do not significantly influence the whole-brain cortical thickness, as correspondingly reported [13]. Another explanation suggests that the effect sizes are too small to detect the influence. Analysing the effects of depression on cortical thickness, critical parameters in neurological alterations are the age of onset and the severity of depression (either recurrent or severity of symptoms) [14]. We could emphasize that moderate to severe depressive symptoms are a relevant factor concerning neurological alteration by being significantly associated with thinner cortices (compared to no symptoms) combined with a history of abuse. Future research could combine these factors to deepen the understanding of the influence of depressive symptoms on cortical thickness.

### The influence of childhood abuse and depressive symptoms on cortical regions

Our results demonstrated a widespread effect of the interaction of childhood abuse and depressive symptoms on cortical thickness. Cortical regions with the highest interaction effects are involved in emotional processing, social cognition, and sensory processing. Cortical thickness of the inferior parietal lobe and the inferior parietal lobe was significantly associated with the childhood abuse-depression-interaction effect in our study. The inferior parietal lobe, divided into the supramarginal gyrus and the angular gyrus, is crucial for overcoming emotional egocentricity avoiding biased social judgments [46]. The inferior parietal lobe was described to handle visuospatial information, be involved in perspective-taking and emotional processing through distancing [47, 48]. Moreover, we found associations with the inferior frontal gyrus, involved in response inhibition and attention control as part of the executive control [49, 50] and previously reported to be thinner in abused participants [51, 52]. Similarly, the cortical thickness of the temporal pole was associated with childhood maltreatment, and physical abuse in particular, before [53, 54]. It is essential in processing social cognition, naming the theory of mind [55]. Furthermore, we found reduced cortical thickness in visual cortex structures, such as the pericalcarine and the later occipital cortex. Earlier studies found reduced grey matter volume, cortical volume, and cortical thickness in the visual cortex in participants with a history of abuse [10, 56–58]. However, associations between these regional reductions and depressive symptoms were not explicitly reported before (e.g., by Schmaal et al. [14]). Thus, we conclude that these reductions show explicitly relevant interaction effects of childhood abuse and depressive symptoms.

### Consequences of childhood abuse on cortical thickness

Every cortical region processes certain qualities of a sensory stimulus [59]. Studies analysing the cellular architecture of the cortex demonstrate that the cortical thickness is contingent on the number of neurons, glia provision, and the neuronal structure [60, 61]. Also, differences in cortical thickness are associated with the structural hierarchical level of processing the stimulus [59]. In the group of participants with no depressive symptoms, the present study found larger cortical thickness in abused compared to non-abused subjects. An explanation for our findings could be the sensitisation theory of Heim et al. (2008) hypothesising that childhood abuse sensitises the pituitary and a counter-adjustment is needed to compensate [62]. In line with this, Teicher et al. [5] argued that maltreated subjects could also differ in brain changes which help them compensate [5]. Previous studies found associations between increased cortical thickness and resilience [73].

Experiencing early life stress, such as childhood abuse, is a severe intrusion in the early development stage [5]. This intrusion can interfere with the neuronal development [63–65]. Consequently, cerebral structures can be prone to future stressful influences [66, 67]. Based on our results, we hypothesise that childhood abuse is a relevant biological disposition that interacts with the current state of psychopathology. Thus, structural brain alterations might follow a different pattern in depressed participants with a history of abuse compared to participants without a history of abuse [5]. Certainly, these results need to be carefully interpreted as other factors such as cognitive training [68], cortical asymmetry [69], and cellular architecture [59, 61] affect the cortical thickness.

Lim et al. [15] argued that the experience of abuse during childhood may lead to a reduction in synapses to protect the child from a hostile environment. These alterations could affect emotional and social processing in depressed patients resulting in a lower treatment outcome. In line with that, meta-analyses revealed that depressed patients with childhood adversity showed reduced benefit from treatment [19, 70]. Future research would be of prime importance to investigate if these abused, and treatment-resistant patients also have remarkably low cortical thickness. Furthermore, the research could focus on how these associations might change with pharmacological or psychotherapeutic therapy and whether these alterations are reversible. We focussed on the consequences of childhood abuse as it is well described in the literature and has a tremendous effect on the cortex [11, 52, 56]. However, it would be important to analyse the interaction effect of neglect and depressive symptoms interacting.

## Limitations

Despite our careful study design, there are several limitations worth noticing. First, we used self-report measures to assess the depressive symptoms and to assess the history of childhood abuse. The PHQ-9 is a validated measurement, but it does not replace a formal psychiatric anamnesis. Also, we focussed only on current depressive symptoms and did not include the influence of lifetime diagnosis of MDD or other psychiatric diagnoses. Therefore, we cannot eliminate other psychiatric diagnoses, which could influence our results. As we did not analyse a psychiatric cohort, we assume that psychiatric symptomatology is low. The CTQ is a self-reported measure that does not assess at which age the abuse or neglect happened. Further, participants with a history of abuse could intentionally not report these traumata due to avoidance or repression.

Second, we focused on abuse-related effects but incorporated participants with the exposure of abuse and neglect. Hence, we cannot differentiate between effects due to childhood abuse or childhood neglect. Childhood abuse and childhood neglect are strongly correlated, and childhood maltreatment is associated with depressive symptoms. However, as we excluded participants exclusively reporting childhood neglect but included participants exclusively reporting childhood abuse, our results are more likely to reflect the effects of childhood abuse. We decided to include subjects with a history of neglect and abuse, as there is a considerable overlap of subjects who experienced both forms of maltreatment [71]. Future studies are needed to differentiate between the effects of abuse and neglect.

Third, the distribution of the abuse and neglect severity is not equally distributed across the depression groups (see Table SI1). As the sample was recruited from the general population and associations between childhood maltreatment and depressive symptoms are well described [19, 20], this is an expected, data immanent issue. Nevertheless, we cannot rule out that this association might impact the results and future studies are needed matching abuse and neglect severity in depressed and non-depressed participants to validate our results.

Fourth, due to the cross-sectional study design, our results do not allow any causal conclusion. Further, although the analysed study sample was large and a large set of potential covariates was included, we did not collect a replication sample. In addition, age-range and sex differences often moderate effects on cortical thickness [72, 73]. We tried to rule out these effects by including both variables as covariates in our statistical models and analysing the results within a sample matched for sex, age, and depression level. We did not adjust for potential comorbidities (such as hypertension or diabetes) as we focus on the general population in which comorbid medical conditions are common. Finally, our study sample is not entirely independent from the ENIGMA consortium, but we included different covariates in our analysis.

## Conclusion

This study investigated the interaction of childhood abuse and depressive symptoms on cortical thickness. Based on a large population-based sample, only in the presence of childhood abuse did depressive symptoms influence the whole-brain cortical thickness. This might explain some of the inconsistencies reported in previous studies. The interaction between childhood abuse and depressive symptoms was associated with a widespread reduction in cortical thickness in regions involved in emotional processing, social cognition, and sensory processing. Larger cortical thickness, in abused subjects without depressive symptoms, might act compensatory and thus reflect resilience against depressive symptoms. Our results support the hypothesis that childhood abuse is moderating the associations between cortical thickness and depressive symptoms, particularly in participants with more severe depressive symptoms. Further research could focus on how different treatments can affect brain alterations associated with childhood abuse and depressive symptoms and whether those are reversible.

## Supplementary Information

Below is the link to the electronic supplementary material.

Supplementary file1 (DOCX 5009 KB)

## Data Availability

SHIP data are applicable via fvcm.med.uni-greifswald.de.
