# Supplementary material for: Interaction of childhood abuse and depressive symptoms on cortical thickness: a general population study
Source: Eur Arch Psychiatry Clin Neurosci. 2022 Feb 25;272(8):1523–34. doi: 10.1007/s00406-022-01387-8 (PMC9653317; doi:10.1007/s00406-022-01387-8)
Supplement: Supplementary file 1 — Supplementary file1 (DOCX 5009 KB) [file 406_2022_1387_MOESM1_ESM.docx]

**Supplementary Information**

[European Archives of Psychiatry and Clinical Neuroscience](https://www.springer.com/journal/406/)

Interaction of childhood abuse and depressive symptoms on cortical thickness: a general population study

Sara Voss^1^, Stefan Frenzel^1^, Johanna Klinger-König^1^, Deborah Janowitz^1,2^, Katharina Wittfeld^1,3^, Robin Bülow^4^, Henry Völzke^5^, Hans J. Grabe^1,3^

^1^ Department of Psychiatry and Psychotherapy, University Medicine Greifswald, Greifswald, Germany

^2^Clinical Centre for Psychiatry und Psychotherapy, Site West, Stralsund, Germany

^3^German Centre for Neurodegenerative Diseases (DZNE), Site Rostock/Greifswald, Greifswald, Germany
^4^Institute for Diagnostic Radiology und Neuroradiology, University Medicine Greifswald, Greifswald, Germany

^5^ Institute for Community Medicine, SHIP/Clinical-Epidemiological Research, University Medicine Greifswald, Greifswald, Germany

Address of correspondence:

Sara Voss; Department of Psychiatry and Psychotherapy, University Medicine Greifswald, Ellernholzstraße 1-2, 17475 Greifswald, Germany. Phone: +49 (0) 3834 86 6842. E-mail: sara.voss@stud.uni-greifswald.de

## Effects of childhood abuse and depressive symptoms as continuous variable on whole-brain cortical thickness

There was a statistically significant two-way interaction between the dichotomous childhood abuse and continuous current depressive symptoms on whole-brain cortical thickness (F(2, 1534) = -2.966, p = 0.003). This result supports the outcome of the categorical depression variable.

## Effects of childhood abuse and depressive symptoms whole-brain cortical thickness with antidepressants as additional covariate

In an additional model, we included the intake of antidepressants (yes/no) as a covariate. 65 participants were on antidepressant medication. Of them, 24 individuals were assorted to the non-depressed group, 21 to the mild depressed, and 20 to the group of moderate to severely depressed. The two-way interaction between childhood abuse and current depressive symptoms on whole-brain cortical thickness remained significant (F(2, 1533) = 5.141, p = 0.007).

| **Table S1** Characteristics of the study sample ordered by the severity of depressive symptoms | | | | | | |
| --- | --- | --- | --- | --- | --- | --- |
|  |  | All subjects  (N = 1551) | Healthy  (*n* = 1064) | Mildly Depressed  (*n* = 397) | Moderately to Severely Depressed  (*n* = 90) | *p*-value ^a^ |
| Sex (female), *n* (%) | | 834 (54%) | 515 (48%) | 259 (65%) | 60 (67%) | < 0.001 |
| Age (years), *M* ± *SD* | | 50 ± 14 | 50 ± 14 | 50 ± 13 | 49 ± 11 | 0.783 |
| BMI, *M* ± *SD* | | 27 ± 4.4 | 27 ± 4.3 | 28 ± 4.6 | 28 ± 4.6 | 0.065 |
| Educational level | |  |  |  |  | < 0.001 |
|  | <10 years, *n* (%) | 167 (10.8%) | 94 (8.8%) | 64 (16.1%) | 9 (10.0%) |  |
|  | =10 years, *n* (%) | 870 (56.1%) | 577 (54.2%) | 231 (58.2%) | 62 (68.9%) |  |
|  | >10 years, *n* (%) | 514 (33.1%) | 393 (36.9%) | 102 (25.7%) | 19 (21.1%) |  |
| Alcohol (g/day), *M* ± *SD* | | 8 ± 11 | 8.3 ± 11 | 7.3 ± 11 | 7.8 ± 12 | 0.199 |
| Smoking | |  |  |  |  | 0.263 |
|  | Never smoker, *n* (%) | 620 (40.0%) | 440 (41.4%) | 151 (38.0%) | 29 (32.2%) |  |
|  | Ex-smoker, *n* (%) | 559 (36.0%) | 383 (36.0%) | 140 (35.3%) | 36 (40.0%) |  |
|  | Current smoker, *n* (%) | 372 (24.0%) | 241 (22.7%) | 106 (26.7%) | 25 (27.8%) |  |
| ICV (dm³), *M* ± *SD* | | 1.587 ± 0.16 | 1.600 ± 0.16 | 1.562 ± 0.15 | 1.544 ± 0.14 | < 0.001 |
| PHQ-9 (summary), *M* ± *SD* | | 3.7 ± 3.5 | 1.9 ± 1.4 | 6.5 ± 1.4 | 13 ± 3.8 | < 0.001 |
| CTQ (summary), *M* ± *SD* | | 32 ± 9.1 | 30 ± 7.5 | 33 ± 9.1 | 39 ± 17 | < 0.001 |
| Abuse severity (summary), *M* ± *SD* | | 17 ± 4.8 | 17 ± 3.7 | 18 ± 5.3 | 21 ± 9.8 | < 0.001 |
| Abuse (yes), *n* (%) | | 122 (7.9%) | 55 (5.2%) | 44 (11%) | 23 (26%) | < 0.001 |
| *Note:* PHQ-9 = Patient-Health-Questionnaire-9; CTQ = Child-Traumatisation-Questionnaire; BMI = Body Mass Index; ICV = Intracranial Volume; *a =* According to one-way ANOVA for continuous or χ^2^-tests for categorical variables to check for possible differences in the groups. | | | | | | |

| **Table S2** Characteristics of the matched sample | | | | | |
| --- | --- | --- | --- | --- | --- |
|  |  | All subjects  (*N* = 240) | Abuse  (*n* = 120) | No Abuse  (*n* = 120) | *p*-value ^a^ |
| Sex (female), *n* (%) | | 68 (28%) | 33 (28%) | 35 (29%) | 0.886 |
| Age (years), *M* ± *SD* | | 50 ± 13 | 50 ± 13 | 50 ± 13 | 0.707 |
| BMI, *M* ± *SD* | | 28 ± 4.8 | 28 ± 4.9 | 28 ± 4.8 | 0.441 |
| Educational level | |  |  |  | 0.595 |
|  | <10 years, *n* (%) | 29 (12%) | 17 (14%) | 12 (10%) |  |
|  | =10 years, *n* (%) | 143 (60%) | 69 (57%) | 74 (62%) |  |
|  | >10 years, *n* (%) | 68 (28%) | 34 (28%) | 34 (28%) |  |
| Alcohol (g/day), *M* ± *SD* | | 5.9 ± 8.6 | 6.1 ± 8.8 | 5.7 ± 8.5 | 0.718 |
| Smoking | |  |  |  | 0.423 |
|  | Never smoker, *n* (%) | 82 (34%) | 41 (34%) | 41 (34%) |  |
|  | Ex-smoker, *n* (%) | 98 (41%) | 45 (38%) | 53 (44%) |  |
|  | Current smoker, *n* (%) | 60 (25%) | 34 (28%) | 26 (22%) |  |
| ICV (dm³), *M* ± *SD* | | 1.559 ± 0.16 | 1.558 ± 0.17 | 1.560 ± 0.15 | 0.905 |
| Depressive Symptoms | |  |  |  | 0.960 |
|  | No, *n* (%) | 108 (45%) | 55 (46%) | 53 (44%) |  |
|  | Mild, *n* (%) | 90 (38%) | 44 (37%) | 46 (38%) |  |
|  | Moderate to severe, *n* (%) | 42 (18%) | 21 (18%) | 21 (18%) |  |
| PHQ-9 (summary), *M* ± *SD* | | 5.8 ± 4.6 | 6.4 ± 5.2 | 5.2 ± 4.0 | 0.045 |
| Neglect (yes), *n* (%) | | 72 (30%) | 72 (60%) | 0 (0%) | < 0.001 |
| CTQ (summary), *M* ± *SD* | | 42 ± 17 | 55 ± 17 | 30 ± 4.1 | < 0.001 |
| Abuse severity (summary), *M* ± *SD* | | 23 ± 9.7 | 30 ± 9.5 | 16 ± 2.0 | < 0.001 |
| *Note:* PHQ-9 = Patient-Health-Questionnaire-9; CTQ = Childhood-Traumatisation-Questionnaire; BMI = Body Mass Index; ICV = Intracranial Volume; *a =* According to one-way ANOVA for continuous or χ^2^-tests for categorical variables to check for possible differences in the groups. | | | | | |

| **Table SI3** Estimated marginal means, standard error, and confidence level (in mm) are displayed for the whole-brain thickness in the whole sample. They were adjusted for sex, age, age*sex-interaction, age², estimated intracranial volume, education, alcohol consumption, smoking, BMI, and waist-to-height ratio | | | | | |
| --- | --- | --- | --- | --- | --- |
| **Factor depression** | **Factor abuse** | **Estimated means for whole-brain thickness** | **Standard error** | **Lower confidence level** | **Higher confidence level** |
| No | No Abuse | 2.339 | 0.013 | 2.314 | 2.364 |
| Mild | No Abuse | 2.340 | 0.013 | 2.314 | 2.366 |
| Moderate to Severe | No Abuse | 2.357 | 0.017 | 2.324 | 2.390 |
| No | Abuse | 2.376 | 0.018 | 2.340 | 2.412 |
| Mild | Abuse | 2.343 | 0.019 | 2.306 | 2.381 |
| Moderate to Severe | Abuse | 2.310 | 0.024 | 2.264 | 2.357 |

| Table SI4 Associations of the interaction of childhood abuse (CA) and depression (Depr) with cortical thickness for the matched sample. Linear regression analyses were adjusted for sex, age, age*sex-interaction, age², estimated intracranial volume, education, alcohol consumption, smoking, BMI, waist size, height, and weight. FDR-p-values are displayed. Significant FDR-p-values are highlighted | | |
| --- | --- | --- |
| Cortical Structure | **Interaction Depr X CA** | **FDR *p-value*** |
| Banks of the superior temporal sulcus | -1.64 | 0.182 |
| Caudal anterior cingulate | -0.15 | 0.909 |
| Caudal middle frontal | -1.51 | 0.198 |
| Cuneus | -1.42 | 0.214 |
| Entorhinal | -0.01 | 0.996 |
| Frontal pole | -1.61 | 0.182 |
| Fusiform | -1.59 | 0.182 |
| Inferior parietal | **-2.89** | **0.043** |
| Inferior temporal | -1.98 | 0.126 |
| Insula | -2.39 | 0.060 |
| Isthmus cingulate | -0.65 | 0.606 |
| Lateral occipital | -2.42 | 0.060 |
| Lateral orbitofrontal | -2.12 | 0.099 |
| Lingual | -1.42 | 0.214 |
| Medial orbitofrontal | -1.61 | 0.182 |
| Middle temporal | -2.17 | 0.096 |
| Paracentral | -0.53 | 0.680 |
| Parahippocampal | -1.09 | 0.349 |
| Pars opercularis | **-2.86** | **0.043** |
| Pars orbitalis | -2.41 | 0.060 |
| Pars triangularis | **-3.52** | **0.018** |
| Pericalcarine | -2.50 | 0.060 |
| Postcentral | -1.62 | 0.182 |
| Posterior cingulate | -1.05 | 0.357 |
| Precentral | -1.09 | 0.349 |
| Precuneus | -1.50 | 0.198 |
| Rostral anterior cingulate | -0.37 | 0.759 |
| Rostral middle frontal | -1.95 | 0.126 |
| Superior frontal | -0.41 | 0.745 |
| Superior parietal | -2.52 | 0.060 |
| Superior temporal | -1.62 | 0.182 |
| Supramarginal | **-2.75** | **0.044** |
| Temporal pole | **-2.83** | **0.043** |
| Transverse temporal | -1.71 | 0.182 |


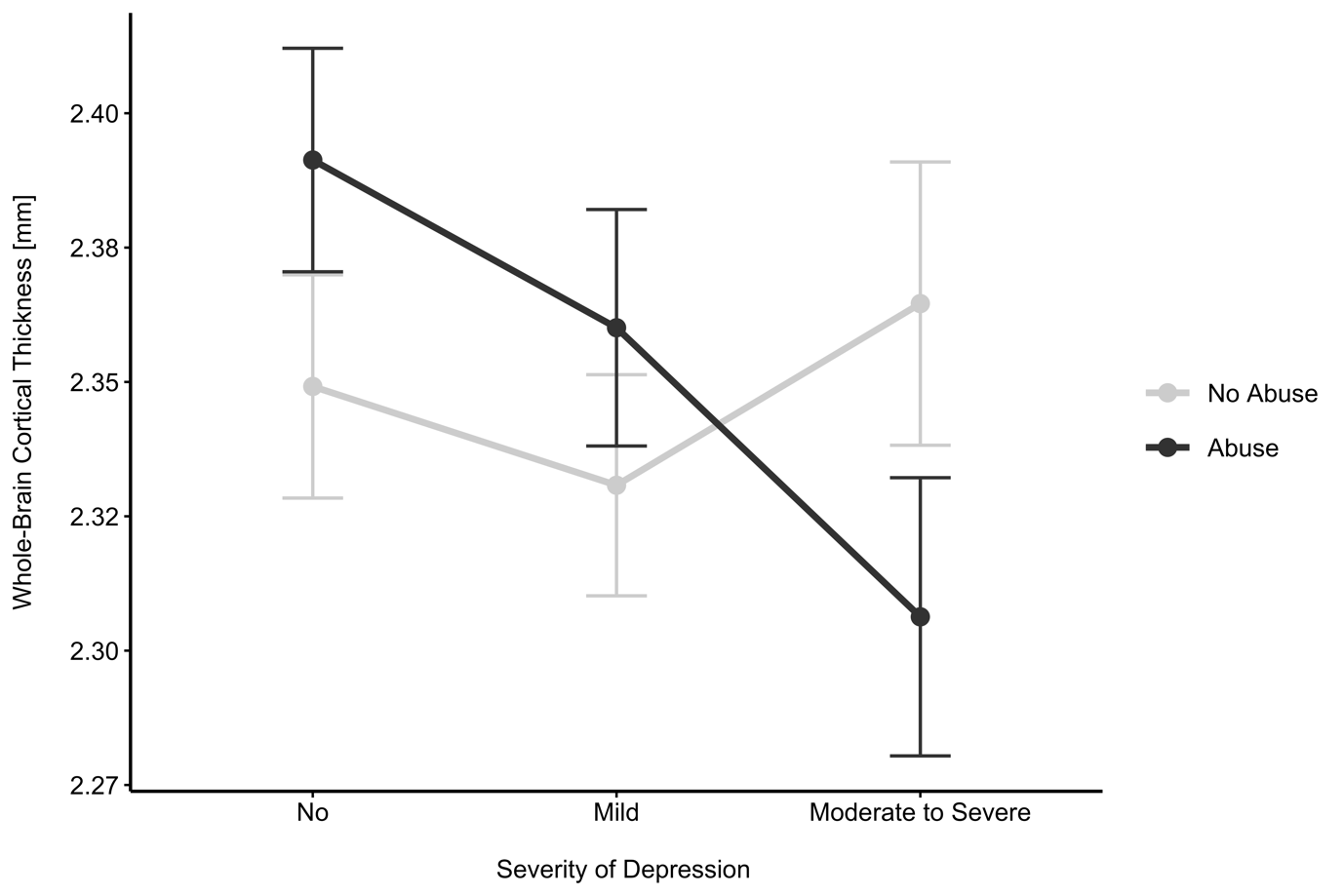


**Fig. SI1** Lineplot showing pairwise comparisons in the matched sample between groups using the estimated marginal means and standard errors of whole-brain cortical thickness across all levels of childhood abuse and depression. In the abused-group (black line), participants with moderate to severe depression had trend significantly thinner whole-brain cortices than non-depressed subjects (t-value = 3.34; p = 0.009)
